# Hydrothermal Generation of Conjugated Polymers Using the Example of Pyrrone Polymers and Polybenzimidazoles

**DOI:** 10.1002/anie.202000367

**Published:** 2020-05-11

**Authors:** M. Josef Taublaender, Stefano Mezzavilla, Sophia Thiele, Florian Glöcklhofer, Miriam M. Unterlass

**Affiliations:** ^1^ Institute of Applied Synthetic Chemistry Technische Universität Wien Getreidemarkt 9/163 1060 Vienna Austria; ^2^ Institute of Materials Chemistry Technische Universität Wien Getreidemarkt 9/165 1060 Vienna Austria; ^3^ Department of Materials Imperial College London, Royal School of Mines Prince Consort Road London SW7 2AZ UK; ^4^ Department of Chemistry and Centre for Plastic Electronics Imperial College London 80 Wood Lane London W12 0BZ UK; ^5^ CeMM – Research Center for Molecular Medicine of the Austrian Academy of Sciences Lazarettgasse 144 1090 Vienna Austria

**Keywords:** green chemistry, high-performance polymers, hydrothermal polymerization, polybenzimidazoles, pyrrone polymers

## Abstract

Various polyimides and polyamides have recently been prepared via hydrothermal synthesis in nothing but H_2_O under high‐pressure and high‐temperature conditions. However, none of the prepared polymers feature a truly conjugated polymer backbone. Here, we report on an expansion of the synthetic scope of this straightforward and inherently environmentally friendly polymerization technique to the generation of conjugated polymers. Selected representatives of two different polymer classes, pyrrone polymers and polybenzimidazoles, were generated hydrothermally. We present a mechanistic discussion of the polymer formation process as well as an electrochemical characterization of the most promising product.

## Graphical abstract

**In hot water**: The scope of hydrothermal polymerization has been expanded. Pyrrone polymers and polybenzimidazoles can now be generated in high‐temperature water by a straightforward one‐step synthesis.

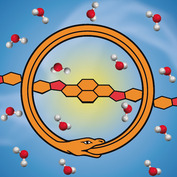

## Introduction

Hydrothermal (HT) reactions have been performed for decades in preparative inorganic chemistry to generate a multitude of different materials ranging from synthetic gemstones to quartz crystals and zeolites.^1^, ^2^, ^3^ However, it has just been in recent years that they were also successfully applied to generate a number of advanced organic high‐performance polymers. To date, only two classes of macromolecular compounds, that is, various polyimides (PIs) and polyamides (PAs), have successfully been generated hydrothermally.^4^, ^5^, ^6^, ^7^, ^8^ The HT preparation of these polymers is characterized by experimental simplicity as well as inherent environmental friendliness and low cost, since neither sophisticated synthetic skills nor toxic solvents nor harmful catalysts are needed. In hydrothermal polymerization (HTP), H_2_O is not only the sole solvent and catalyst, it is also the only reaction byproduct formed (PIs and PAs are condensation polymers). Hence, the handling and disposal of hazardous substances can be greatly minimized.

**Scheme 1:**
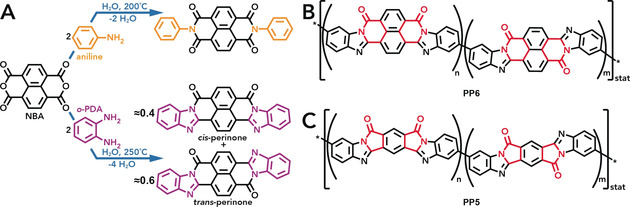
HT cyclocondensations: A) Reaction of aniline and NBA to form a naphthalene bisimide (top) and reaction of *o*‐PDA and NBA to form a mixture of *cis*‐ and *trans*‐perinone (bottom). B) PP6: A six‐membered cyclic amide linkage (red) exists between the two comonomers. C) PP5: The comonomers are linked via a five‐membered cyclic amide (red). Both PP5 and PP6 display two possible configurations of r.u.s (*cis* and *trans*).

We have strong indication to believe that, besides the outstanding properties of high‐temperature water (HTW), a substantial energetic driving force—for example, generated through the formation of strong bonds (i.e. bonds of high dissociation energy), cyclization reactions, conjugation, and/or crystallization—is beneficial for a polymerization to proceed under HT conditions. For example, when a suitable aromatic anhydride group (or the corresponding dicarboxylic acid) such as in naphthalene bisanhydride (NBA) is reacted with a primary amine (e.g. aniline), a cyclic imide moiety is formed via cyclocondensation (Scheme 1 A top).^9^ Generally, the HT formation of imides has already been extensively investigated: Both small molecules^9^ and a multitude of different PIs^4^, ^5^, ^6^, ^8^, ^10^ can be efficiently synthesized. In the example introduced above, the formed imide moieties are cyclic and six‐membered. Furthermore, the imide's C=O groups and its N atom are linearly conjugated. However, the entire imide group is only cross‐conjugated with the naphthalene moiety. It is generally known that the electronic communication between cross‐conjugated groups is significantly weaker than that in linearly conjugated groups.^11^ Moreover, cross‐conjugation also leads to a drastically reduced conductance and, hence, negatively affects charge transport properties.^12^ However, when instead of an aromatic monoamine group (to form an imide) an aromatic *o*‐diamine moiety is reacted with an aromatic anhydride moiety (as in NBA; Scheme 1 A bottom), a double cyclization can occur. Consequently, an imidazole group fused to a cyclic amide function is generated, which is an even stronger linking function than an imide.^13^ Additionally, by connecting the starting compounds via double cyclization, linear conjugation between the building blocks can be achieved. However, such a HT double cyclization has not yet been reported for generating a polymer. There is just one report on synthesizing a small molecule via HT double cyclization: reacting *o*‐phenylene diamine (*o*‐PDA) with NBA yields an isomeric mixture of the carbonyl dye perinone (Scheme 1 A bottom).^13^ As a consequence of perinone's highly aromatic and conjugated structure, the molecule is fully planar. Planarity, aromaticity, and conjugation impart properties such as extreme thermal and chemical stability and it would clearly also be highly interesting to implement these features in an organic polymer. Generally, by applying this perinone‐type linking chemistry and reacting suitable aromatic bis(*o*‐diamine)s with aromatic dianhydrides (or their tetracarboxylic acids), the corresponding pyrrone polymers (PPs, Scheme 1 B,C) can be obtained. These materials were intensively investigated during the 1960s for their high *T*‐ and radiation resistance, which was considered as promising for the aerospace sector.^14^, ^15^ More recently, PPs have attracted attention for electronics and optics applications (especially for nonlinear optical properties) due to their conjugated nature.^16^, ^17^, ^18^, ^19^ Furthermore, considering that PIs and quinone‐decorated polymers have been studied as aqueous battery electrodes in neutral and acidic electrolytes,^20^, ^21^, ^22^ we anticipated that PPs could exhibit useful electrochemical properties in aqueous electrolytes as well. Just like HTP, aqueous batteries take advantage of the environmentally benign nature of H_2_O.^23^

Taking inspiration from our recent reports on the HTP of various PIs as well as the HT preparation of perinone, we have now set out to both expand the number of hydrothermally obtainable polymers and to generate polymers with a conjugated backbone by HTP. We consider this as a truly crucial step towards lifting HTP to a new level of broader applicability. The two most prominent representatives of PPs are shown in Scheme 1 B,C. Note that the type of dianhydride employed determines the size of the cyclic amide moiety linking the two comonomers. While NBA leads to a six‐membered cyclic amide, pyromellitic dianhydride (PMDA) gives rise to a five‐membered cyclic amide moiety. The corresponding semiladder‐type PPs will herein be referred to as PP6 (aka BBB) and PP5, respectively. In general, a random distribution of *cis* and *trans* configurations of repeating units (r.u.s) along the polymer chain is expected. The two common synthetic procedures for PPs are: (*i*) controlled and stepwise heating of the neat comonomers in polyphosphoric acid yielding PP particles,^14^, ^15^ which can be processed by high‐*T* molding;^24^ (*ii*) stirring the neat comonomers at room temperature (rt) in an aprotic, polar solvent such as *N*,*N*‐dimethylformamide (DMF) to obtain a processable poly(amide amino acid) solution, which can be processed into, for example, films and fibers before a final thermal curing step at *T*≥300 °C yields PP.^25^, ^26^ Clearly, these latter procedures are tedious and far from being environmentally benign. Therefore, we were aiming at a facile, green synthetic strategy towards PPs in nothing but “hot water”.

## Results and Discussion

### HTP for Synthesis of PP6

**Scheme 2:**
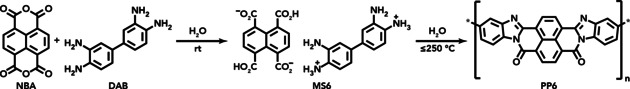
Synthesis of PP6: First, NBA hydrolyzes to NTCA which reacts with DAB to give MS6 (position of charges in the depiction of MS6 is arbitrary). Second, when MS6 is subjected to HT conditions, the double cyclization to form PP6 readily takes place.

When investigating the general feasibility of hydrothermally synthesizing PPs, we attempted to keep the initially used comonomers as similar as possible to the precursors of perinone, and also applied the reaction conditions that had been found to be optimal for HT perinone formation.^13^ Hence, NBA and 3,3′‐diaminobenzidine (DAB) were used to attempt PP6 synthesis under HT conditions. Since the use of monomer salts (MSs) as precursors instead of working with neat comonomers has proven to be highly beneficial for the HTP towards PIs,^4^, ^5^, ^6^ we intended to prepare a MS of DAB and naphthalene tetracarboxylic acid (NTCA; from hydrolysis of NBA) as the precursor for PP6 (Scheme 2). MSs often intrinsically provide equimolar comonomer stoichiometry, which is of prime importance for obtaining high‐molecular‐weight products by a polycondensation (Carother's Law). Moreover, compared to neat amines, MSs feature—at least partially—protonated NH_2_ groups, which are more stable towards oxidation and hence storable for much longer periods of time without special precautions. Additionally, MSs can be expected to exhibit altered reactivity and solubility compared to neat comonomers due to their ionic nature, preorganization in a salt crystal, and hence close spatial proximity of reactive groups already in the solid state.^27^

**Figure 1:**
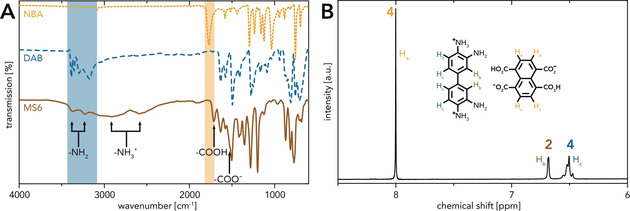
Characterization of MS6: A) ATR‐FTIR spectra of MS6 and its starting compounds DAB and NBA: characteristic modes of the starting compounds are highlighted by colored boxes; modes indicative for MS6 are designated by arrows. B) ^1^H NMR spectrum of MS6 measured in [D_6_]DMSO: an integral ratio H_a_:H_b_:H_c_=4:2:4 confirms a 1:1 molar ratio of NTCA:DAB.

The MS suitable for synthesizing PP6 (abbreviated as MS6) was generated by precipitation in H_2_O (see the Supporting Information). MS6 was obtained as a purple solid. Analysis via attenuated total reflectance Fourier transform infrared (ATR‐FTIR) spectroscopy and ^1^H NMR spectroscopy confirmed its successful formation. The ATR‐FTIR spectra of MS6 and its starting compounds NBA and DAB clearly differ from each other (Figure 1 A). The modes characteristic for the starting compounds (highlighted by colored boxes; ν_C=O_(NBA)≈1770 cm^−1^, ν_N‐H_(DAB)≈3450–3200 cm^−1^)^28^, ^29^ are absent in the MS6 spectrum. Instead, several modes indicative for the formation of MS6 (highlighted by arrows; ν_N‐H_(NH_2_)≈3375 cm^−1^, ≈3220 cm^−1^; ν_N‐H_(NH_3_^+^)≈2885 cm^−1^, ≈2585 cm^−1^; ν_C=O_(COOH)≈1710 cm^−1^; ν_C=O_(COO^−^)≈1525 cm^−1^) are observed. Figure 1 B shows the ^1^H NMR spectrum of MS6: The singlet (H_a_) at 8.00 ppm corresponds to the naphthalene protons, whereas the singlet (H_b_) at 6.65 ppm and the multiplet (H_c_) at 6.52 ppm stem from the biphenyl protons. The integral ratio H_a_:H_b_:H_c_=4:2:4 confirms a 1:1 DAB:NTCA composition.

**Figure 2:**
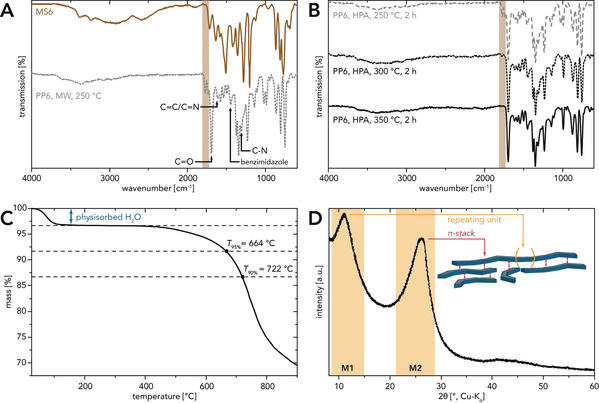
Characterization of PP6: A) ATR‐FTIR spectra of PP6 (MW, *c*=0.01 mol L^−1^, *T*_R_=250 °C, *t*_H_=10 min, *t*_R_=15 min) and its precursor MS6: characteristic PP6 modes are highlighted by arrows; anhydride end‐groups are indicated by a brown box. B) ATR‐FTIR spectra of PP6 (HPA, *c*=0.01 mol L^−1^, *t*_R_=2 h) prepared at different *T*_R_s: with increasing *T*_R_ the anhydride end‐group modes (brown box) gradually disappear. C) TGA curve (N_2_, 10 °C min^−1^) of a fully condensed PP6 sample (HPA, *c=*0.01 mol L^−1^, *T*_R_=350 °C, *t*_R_=2 h): an initial mass loss stems from physisorbed H_2_O; polymer degradation temperatures *T*_95 %_ and *T*_90 %_ are indicated. D) PXRD patterns of PP6 (HPA, *c*=0.01 mol L^−1^, *T*_R_=350 °C, *t*_R_=2 h): two broad features are found—M1 (length of r.u.) and M2 (interchain distance).

Subsequently, a microwave (MW)‐assisted HTP towards PP6 using MS6 as precursor was carried out. For this, an aqueous dispersion of MS6 (*c*=0.01 mol L^−1^) was placed in a glass liner that was transferred into a PTFE‐lined stirred MW autoclave, and heated via MW irradiation within a heating time (*t*_H_) of 10 min to the desired reaction temperature (*T*_R_) of 250 °C. *T*_R_ was kept constant for a reaction time (*t*_R_) of 15 min. After cooling back to rt, the glass liner contained two different distinct layers: a solid, black sediment at the bottom and a translucent, colorless, clear liquid supernatant. The black color of the solid was already indicative of PP6 formation. ATR‐FTIR spectroscopy of the solid (Figure 2 A) confirmed a successful double cyclization through the presence of several characteristic PP6 modes,^15^, ^30^ which are similar to those of perinone:^13^ ν_C=O_(PP6)≈1700 cm^−1^; ν_C=C/C=N_(PP6)≈1620 cm^−1^ (combined mode); ν(benzimidazole)≈1450 cm^−1^ (in‐plane vibration); ν_C‐N_(PP6)≈1310 cm^−1^. Yet, intense modes occurring at ≈1780 cm^−1^ and ≈1740 cm^−1^ (Figure 2 A, brown box) point to the presence of anhydride end‐groups.^31^, ^32^ Clearly, free anhydride groups imply that also unreacted NH_2_ moieties must exist in the generated polymer. However, the N‐H stretching modes are expected to be inherently weaker than the anhydride end‐group modes. Intermolecular H‐bonding with PP6’s C=O groups produces significant band broadening and additionally lowers the mean absorption frequency.^33^ Hence, only the indicative and well‐pronounced anhydride C=O modes are used here and in the following to estimate the success of a HTP experiment. Based on the intensity of these C=O modes, we expect PP6 generated in this initial test experiment to be of relatively low molecular weight. In further consequence, various attempts (see the Supporting Information) were made in order to obtain products of higher molecular weight: neither lowering *c*, altering the pH, increasing *t*_R_, extending *t*_H_, nor the addition of a non‐nucleophilic base (which had been shown to promote HT imide formation)^9^ led to a decrease in anhydride end‐group mode intensities in the ATR‐FTIR spectra. All these HTP experiments were carried out using the conventional, stirred MW‐assisted setup at *T*_R_=250 °C (which is our setup's maximum continuous operation temperature). However, investigating *T*_R_s≥250 °C seemed to be another promising strategy for obtaining products of higher molecular weight. Upon elevating *T*, the static dielectric constant of H_2_O continuously decreases,^34^ which allows for a significantly better dissolution of various organic compounds (especially aromatics) that are virtually insoluble in H_2_O under ambient conditions.^35^, ^36^, ^37^ Furthermore, classical PP synthesis also uses *T*_R_s≥300 °C.^26^ For employing higher *T*_R_s, we had to switch our experimental setup to a nonstirred high‐temperature high‐pressure autoclave (referred to as HPA; see the Supporting Information for details). The sample preparation procedure remained the same as for the MW‐assisted experiments plus 10 bar of Argon pre‐pressure was applied prior to heating.^71^

In the following, HTPs of MS6 at various *T*_R_s (250 °C, 275 °C, 300 °C, 325 °C, 350 °C) and *t*_R_s (15 min, 2 h, 12 h) were carried out. Initial experiments at *T*_R_=250 °C immediately showed that the absence of stirring in the HPA makes longer *t*_R_s compared to the MW‐assisted stirred setup necessary for achieving the same results. Furthermore, at a constant *t*_R_ the anhydride end‐group modes in the ATR‐FTIR spectra diminished in intensity with increasing *T*_R_ (Figure 2 B). While at *t*_R_=2 h the modes are still visible for *T*_R_s up to 325 °C, they have entirely vanished for *T*_R_=350 °C. For *T*_R_s of 250 °C and 275 °C, respectively, prolonging *t*_R_s did not lead to a decrease in intensity of the end‐group modes. However, for *T*_R_s of 300 °C and 325 °C an extension of the *t*_R_ to 12 h resulted in the complete disappearance of anhydride end‐group modes. Moreover, for none of the samples were any modes found in the ATR‐FTIR spectra indicating incomplete cyclization or nonlinear branching (e.g. amide or imide).^26^, ^31^ Based on these results, we conclude that fully condensed, linear PP6 of different degrees of polymerization can be intentionally synthesized hydrothermally without the need for any cosolvents or condensation catalysts simply by adjusting *T*_R_ and *t*_R_.

We then investigated the thermal stabilities of such fully condensed PP6 samples via thermogravimetric analysis (TGA; Figure 2 C). An initial mass loss between rt and 120 °C, present in all samples, is attributed to physisorbed H_2_O. It is well known that PP6 always contains a certain amount of physisorbed H_2_O due to H‐bonding to the lone‐pair‐bearing heteroatoms (N, O) in the polymer.^38^, ^39^ Taking the initial mass loss due to physisorbed H_2_O into account, the temperature of 5 % mass loss (*T*_95 %_) is 664 °C, and the temperature of 10 % mass loss (*T*_90 %_) is 722 °C. These high values are in accordance with the literature data for fully condensed PP6.^15^, ^26^, ^31^ Furthermore, it was also possible to dissolve PP6 in methanesulfonic acid, which allowed for the fabrication of thin films following a procedure recently described in the literature.^40^ UV/Vis absorption spectra of diluted solutions as well as of the fabricated thin films (see the Supporting Information) agree well with previous reports and indicate the generation of a fully conjugated polymer backbone.^41^

For linear PIs and PA networks, HT preparation gives rise to outstandingly high crystallinity superior to that of conventionally prepared analogues.^4^, ^7^ To date, exclusively amorphous PPs have been prepared. Powder X‐ray diffraction (PXRD) measurements of fully condensed PP6 from HTP are entirely identical for all samples (representative diffractogram in Figure 2 D). The pattern only contains two broad features: one with a maximum at 11.6° (2*θ*, Cu‐K_α_) (labeled M1), and one with a maximum at 25.8° (2*θ*, Cu‐K_α_) (labeled M2). While M2 corresponds to the interchain distance (*n*=1; *d*_hkl_(25.8°)≈3.5 Å) which originates from interchain π‐stacking between the completely planar r.u.s, M1 can be assigned to the length of the r.u. (*n*=2; *d*_hkl_(11.6°)≈15.2 Å). These observations agree with the literature.^42^, ^43^ In contrast to PIs and PAs from HTP,^4^, ^7^ PP6 features no improved crystallinity compared to classically prepared PP6. Attempts to increase PP6’s crystallinity by performing HTPs using lower *c*(MS6) were not successful (see the Supporting Information). However, the highly ordered PA networks reported by Stewart et al. were obtained by devitrification of initially prepared amorphous precursor networks based on the reversibility of amide bonds under HT conditions.^7^ PA devitrification requires *T*_R_s of 240–250 °C and *t*_R_s of 3–7 days.^7^ Inspired by this report, we resubjected our PP6 samples to HT conditions at different *T*_R_s (250 °C, 300 °C) for 7 days hoping that also for PP6 an increase in crystallinity could be realized through similar bond reversibility. Unfortunately, no changes were observed in the PXRD patterns (see the Supporting Information). We believe that the lack of reversibility must be related to the fact that a perinone‐type linking is much more stable, linearly conjugated, and significantly less prone to hydrolysis than an amide linking. As ATR‐FTIR spectra of PP6 before and after devitrification treatment were virtually identical, we conclude that PP6 is fully stable towards prolonged exposure to “hot H_2_O”.

**Figure 3:**
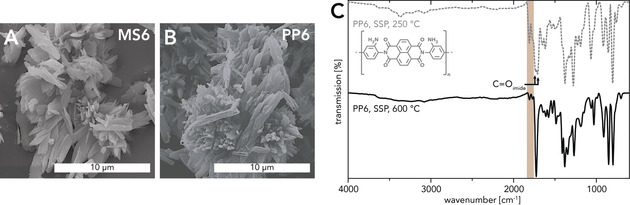
SEM image of MS6 (A) and PP6 (HPA, *c*=0.01 mol L^−1^, *T*_R_=350 °C, *t*_R_=2 h) (B). C) ATR‐FTIR spectra of PP6 generated via SSP at *T*_R_=250 °C (*t*_R_=2 h) and *T*_R_=600 °C (*t*_R_=30 min): the 600 °C sample shows identical modes compared to HT PP6 including anhydride end‐groups (brown box), while SSP at 250 °C leads to imide modes (arrows) and intense anhydride end‐group modes (brown box).

For hydrothermally prepared linear PIs, a MS dissolution–polymerization–polymer crystallization mechanism has been shown, and thus the transformation from the corresponding MS precursor to the PI is accompanied by a significant change in morphology.^4^, ^44^ In contrast, scanning electron microscope (SEM) images of MS6 and PP6 revealed that their morphologies are in fact strikingly similar: Both are mainly composed of microsheets and needles (≈2–10 μm in length) that are agglomerated into bigger structures (Figure 3 A,B). Most interestingly, this shape retention from MS6 to PP6 is found for all samples irrespective of the reaction conditions. Thus, we conclude that the conversion of MS6 to PP6 must mainly take place in the solid state, while dissolution or melting do not seem to occur. It is well known for various types of condensation polymers (PAs, polyesters, PIs) that the heat treatment of suitable MS precursors can lead to solid‐state polymerization (SSP) with retention of the MS's shape.^45^, ^46^ Such SSPs are typically performed under solvent‐free conditions by heating the MS to a *T* below its melting point.^47^ Yet, reports on SSP in dispersed media exist,^48^, ^49^ and for PIs, SSP during HTP is a possible reaction pathway that occurs to varying extent depending on the MS solubility in HTW and its SSP temperature.^5^, ^10^ Here, however, the observation that MS6 and PP6 morphologies are virtually identical strongly points to the transformation basically exclusively occurring via SSP. This rather unexpected finding raised the question whether the presence of H_2_O is at all necessary for the transformation of MS6 to PP6 or whether just heating MS6 to *T_R_*s≥250 °C would be sufficient. Thus, we performed several SSPs, heating neat MS6 to different *T*_R_s (250 °C, 350 °C, 600 °C) under N_2_ atmosphere. Intriguingly, none of the SSP experiments yielded fully condensed PP6. (*i*) SSP at 250 °C (*t*_R_=2 h, 12 h) gave low‐molecular‐weight imide intermediates with anhydride end‐groups (ν_C=O_(imide)≈1710 cm^−1^, ≈1675 cm^−1^; ν_C=O_(anhydride)≈1780 cm^−1^, ≈1740 cm^−1^; Figure 3 C and the Supporting Information). Interestingly, we never found such imides in any of the HTPs towards PP6. Note that *T*_R_=250 °C was already sufficient for HT PP6 formation. (*ii*) SSP at 350 °C (*t*_R_=2 h) led to some perinone‐type linking, but significant amounts of imide and anhydride‐end group modes were still present (see the Supporting Information). (*iii*) ATR‐FTIR analysis of a last experiment performed at 600 °C (*t*_R_=30 min; Figure 3 C) clearly confirmed the generation of a PP6 product no longer containing residual imide moieties. However, anhydride end‐groups are still visible, indicating a low degree of polymerization. This nicely aligns with observations by Morgan and Scott, who found that full condensation of MS6 to PP6 was only reached at ≈630 °C.^50^ However, in this *T*‐regime decomposition already sets in, which limits the attainable degrees of polymerization.

This SSP study strongly suggest that H_2_O plays a crucial role during the transformation of MS6 to PP6. First, HTW significantly lowers the necessary *T*_R_s for obtaining PP6; second, it makes it possible to generate products of higher molecular weight; and third, it additionally has a strong influence on the mechanism of the transformation and facilitates the double cyclization towards PP6. In principle, several possible explanations are conceivable: (*i*) The increased ionic product of H_2_O under HT conditions^51^ could promote the cyclocondensation.^52^ This would have to involve a migration of H^+^ (H_3_O^+^) and/or OH^−^ into the solid MS particles. Note that for many (organic) materials—for example, aqueous dispersions/solutions of solid biomass, graphene oxide, or sugars—it is known that HT treatment can significantly facilitate dehydration reactions.^53^, ^54^, ^55^ (*ii*) The autogenously arising pressure when H_2_O is heated in an autoclave could play a beneficial role in the transformation. There are several literature reports demonstrating the feasibility of reactive hot pressing towards PPs starting from either properly mixed neat comonomers or MSs as precursors.^50^, ^56^ However, *T_R_*s of ≈450 °C and pressures of at least 275 bar had to be applied in order to achieve full cyclization and dense products. This compares to pressures of ≈165 bar in HTP at 350 °C.

### HTP for Synthesis of PP5

**Figure 4:**
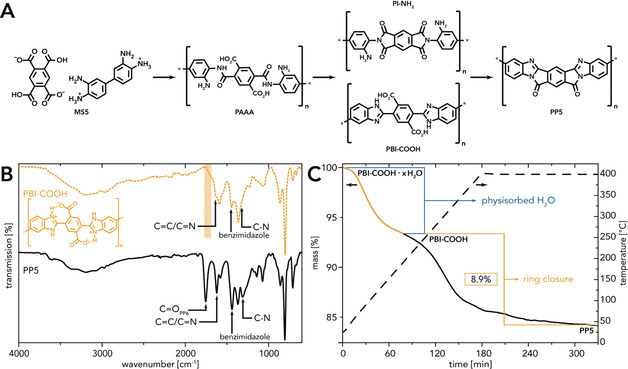
Identification of PBI‐COOH and PP5: A) Overview of possible intermediates. B) ATR‐FTIR spectra of PBI‐COOH (MW, *c*=0.01 mol L^−1^, *T*_R_=250 °C, *t*_H_=10 min, *t*_R_=15 min) and PP5 synthesized via heating of solid PBI‐COOH (*T*_R_=400 °C, *t*_R_=2 h, N_2_ atmosphere): characteristic modes are indicated by arrows and the ν_C=O_(COOH) region is highlighted by an orange box. C) TGA curve (N_2_, 2 °C min^−1^) of PBI‐COOH (MW, *c*=0.01 mol L^−1^, *T*_R_=250 °C, *t*_H_=10 min, *t*_R_=15 min) evincing the transformation to PP5.

**Scheme 3:**
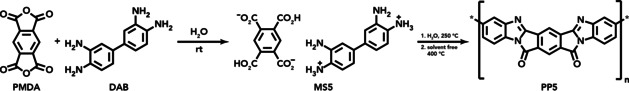
Synthesis of PP5: First, PMDA hydrolyzes to PMA which reacts with DAB to give MS5 (position of charges in the depiction of MS5 is arbitrary). Second, when MS5 is subjected to HT conditions followed by thermal treatment (400 °C, solvent‐free) of the isolated intermediate, PP5 is formed.

After the HT generation of PP6 had been achieved, we decided to also attempt generating the more strained five‐membered amide linkages, that is, the HTP of PP5 (Scheme 3). Therefore, we first prepared a suitable MS (MS5; see the Supporting Information) of pyromellitic acid (PMA) and DAB. The successful formation of MS5 including 1:1 stoichiometry of PMA:DAB was confirmed by ATR‐FTIR and ^1^H‐NMR analysis (see the Supporting Information). Surprisingly, when MS5 was subjected to HT conditions using the conditions already applied for PP6 synthesis (MW, *c*=0.01 mol L^−1^, *T*_R_=250 °C, *t*_H_=10 min, *t*_R_=15 min) the reaction yielded a fine, dark orange powder topped by a translucent liquid phase. This aspect lies in stark contrast to the deep black color reported for PP5.^25^ Furthermore, the corresponding ATR‐FTIR spectrum (Figure 4 B; top curve) lacked the intense C=O mode at ≈1755 cm^−1^ characteristic for PP5. Nevertheless, the spectrum is still significantly different from that of MS5 (e.g. disappearance of NH_2_ and NH_3_^+^ modes; see the Supporting Information). Yet, neither the well‐defined ATR‐FTIR spectrum, nor the product's appearance indicate an uncontrolled decomposition. We considered it more likely that a distinct chemical reaction had taken place that, however, did not yield PP5. In accordance with the literature on similar compounds,^57^, ^58^, ^59^ three structural possibilities seemed probable (Figure 4 A): (*i*) An initial condensation might generate a poly(amide amino acid) (PAAA). In a second condensation step two different species could form next: (*ii*) a poly(imide amine) (PI‐NH_2_) or (*iii*) a poly(benzimidazole acid) (PBI‐COOH). In theory, a mixture of some or even all of these different species, as well as a crosslinked network (necessarily implying amide linkages) is conceivable. Yet the product's ATR‐FTIR spectrum shows neither amide nor imide modes. Hence, the formation of PAAA, PI‐NH_2_, or an amide‐crosslinked network can be excluded. Moreover, the combined C=C/C=N ring vibration mode at ≈1620 cm^−1^, the benzimidazole in‐plane vibration mode at ≈1445 cm^−1^, and the C‐N stretching mode at ≈1315 cm^−1^ are found. These modes are strongly indicative of polybenzimidazoles (PBIs),^30^ and also in accordance with a low‐molecular‐weight model compound synthesized from PMDA and *o*‐PDA.^58^ Therefore, we expected the material obtained via HT treatment of MS5 to be PBI‐COOH. Interestingly, the ATR‐FTIR spectrum does not feature a C=O mode of ‐COOH (characteristic region indicated by orange box in Figure 4 B), which we attribute to an intramolecular proton transfer generating H‐bonded imidazolium and carboxylate groups (cf. structure in Figure 4 B).^58^ However, for simplicity we will here refer to the obtained material as PBI‐COOH.

To transform PBI‐COOH to PP5, just one additional condensation–cyclization step is required. However, all our attempts to hydrothermally achieve this final ring closure to a significant extent were unsuccessful (see the Supporting Information). Interestingly, upon elevating *T_R_* for HT PP5 synthesis, we found that *T_R_*s≥300 °C (experiment performed in HPA) lead to decomposition. This lies in stark contrast to PP6, where only HTPs at such *T_R_*s allow for synthesizing high‐molecular weight products. In fact, we speculate that the intramolecular H‐bonds between imidazolium and ‐COO^−^ (which we expect to be seven‐membered cyclic motifs) energetically stabilize PBI‐COOH (especially in a protic environment such as HTW) and hence make the final ring closure more difficult, if not hydrothermally impossible. Having demonstrated for the case of MS6 that different reactivities during HTP and SSP occur, we decided to investigate post‐polymerization solid‐state heat treatment of PBI‐COOH for triggering its transformation towards PP5. Luckily, this solvent‐free heat treatment indeed enabled the conversion to PP5: When PBI‐COOH was heated to 400 °C under N_2_ atmosphere, the desired second ring closure could be achieved. ATR‐FTIR analysis (Figure 4 B; bottom curve) is nicely in accordance with the literature and confirms PP5 formation.^14^, ^60^ All characteristic modes are present: ν_C=O_(PP5)≈1755 cm^−1^; ν_C=C/C=N_(PP5)≈1620 cm^−1^ (combined mode); ν(benzimidazole)≈1440 cm^−1^ (in‐plane vibration); ν_C‐N_(PP5)≈1310 cm^−1^). Modes indicating incomplete ring closure (amide, imide, amino, carbonyl/carboxyl) are absent.

**Figure 5:**
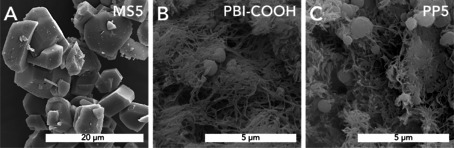
SEM image of MS5 (A), PBI‐COOH (MW, *c*=0.01 mol L^−1^, *T*_R_=250 °C, *t*_H_=10 min, *t*_R_=15 min) (B), and PP5 synthesized by solid‐state heat treatment of PBI‐COOH (*T*_R_=400 °C, *t*_R_=2 h, N_2_) (C).

Subsequently, a TGA experiment was performed to monitor the reaction from PBI‐COOH to PP5 (Figure 4 C). An initial mass loss of ≈7 % is attributed to physisorbed H_2_O—a well‐known phenomenon for various PBIs and PPs.^39^, ^61^ The second mass loss step of ≈8.9 % perfectly matches the calculated m(H_2_O) for full condensation through the desired ring‐closure (see the Supporting Information). PXRD measurements (see the Supporting Information) evinced that PP5 as well as PBI‐COOH are amorphous, featuring only one broad halo centered around ≈26° (2*θ*, Cu‐K_α_) indicating low order yet intermolecular π‐stacking interactions between the polymer chains. SEM measurements of MS5, PBI‐COOH and PP5 (Figure 5) revealed strong morphological similarities between PBI‐COOH and PP5, indicating the absence of any softening phenomena during the corresponding transformation. Both contain mainly mats of fibroids and a few spherical particles (≈0.5–1 μm in diameter), while MS5 is exclusively composed of angular particles of ≈5–10 μm. These morphologies imply that the HTP towards PBI‐COOH must occur via a dissolution—polymerization–precipitation mechanism, whereas the second solvent‐free reaction step from PBI‐COOH to PP5 proceeds in the solid state.

Overall, PP5 and PP6 differ significantly in their synthesis. The fact that isolable PBI‐COOH is obtained when MS5 is subjected to HT conditions suggested that also pure, nonfunctionalized PBIs might be available by HTP.

### HTP for Synthesis of PBI

**Figure 6:**
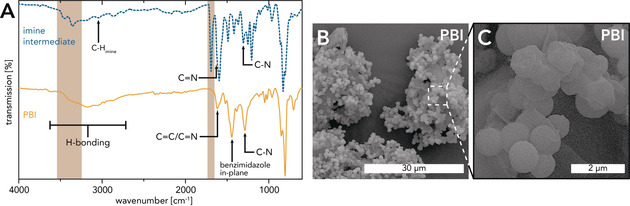
A) ATR‐FTIR spectra of imine intermediate obtained by stirring the comonomers in H_2_O at rt (*c*=0.01 mol L^−1^, *t_R_*=15 min) and PBI generated from imine intermediate (MW, *c*=0.01 mol L^−1^, *T_R_*=250 °C, *t_H_*=10 min, *t_R_*=15 min): broad modes in the range of 3600–2800 cm^−1^ arise from intermolecular H‐bonding either between different imidazole moieties or between an imidazole group and physisorbed H_2_O.^65^ B,C) SEM images of PBI (MW, *c*=0.01 mol L^−1^, *T_R_*=250 °C, *t_H_*=10 min, *t_R_*=15 min).

**Scheme 4:**
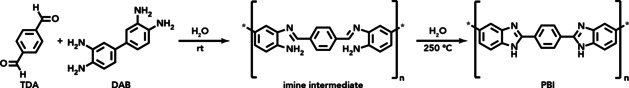
Synthesis of PBI: Initially, TDA and DAB form an imine intermediate (locations of imine linkages are displayed arbitrarily). Subjecting the imine intermediate to HT conditions generates PBI.

In analogy to the HTP of PIs^4^, ^5^, ^6^, ^44^ and PPs (this work), we first attempted to prepare a MS from terephthalic acid (TA) and DAB. However, the preparation of the corresponding MS could not be achieved, which we explain by the fact that the p*K*_a_ difference between TA (p*K*_a_(TA)=3.49) and DAB (p*K*_a_(DAB)=4.39) is too small for salt formation.^62^ Therefore, we aimed at preparing PBI via directly subjecting the neat comonomers TA and DAB suspended in H_2_O to HT conditions (MW, *c*=0.01 mol L^−1^, *T_R_*=250 °C, *t_H_*=10 min, *t_R_*=15 min). A brown powder at the bottom of the liner was obtained. As ATR‐FTIR measurements did not confirm PBI formation, we assumed that TA lacks sufficient reactivity. Hence, we decided to replace TA by a more reactive carbonyl compound: inspired by Neuse et al. we chose to use terephthalaldehyde (TDA).^63^ They reported the preparation of an imine prepolymer generated under anaerobic conditions at *T*_R_≤25 °C in an aprotic, polar solvent, and its subsequent conversion to PBI upon heating to 60 °C in the presence of a transition metal catalyst under aerobic conditions. Instead, our chosen synthetic strategy (Scheme 4) requires neither oxygen exclusion nor transition metal catalysts. The only solvent and catalyst employed is H_2_O. When stirring an aqueous dispersion of TDA and DAB at rt, we observed a rapid color change (from beige to red) of the solution as well as the formation of a red solid precipitate. ATR‐FTIR spectroscopy (Figure 6 A) allowed for identifying the precipitate as a low‐molecular‐weight oligoimine intermediate. In addition to the characteristic imine modes (arrows in Figure 6 A; ν_C‐H_(imine)≈2860 cm^−1^; ν_C=N_(imine)≈1615 cm^−1^; ν_C‐N_ (imine)≈1295 cm^−1^),^63^, ^64^ also intense aldehyde (ν_C=O_ (aldehyde)≈1695 cm^−1^; brown box) and amino (ν_N‐H_(NH_2_)≈3450 cm^−1^, ≈3355 cm^−1^; brown box) modes are found. Such imine intermediates were already observed by Neuse et al.^63^ Subsequent HT treatment of the aqueous red dispersion (MW, *c*=0.01 mol L^−1^ *T_R_*=250 °C, *t*_H_=10 min, *t*_R_=15 min) yielded an orange solid topped by a translucent, clear supernatant and ATR‐FTIR measurements (Figure 6 A) evinced the transformation of the intermediate to PBI. Clearly, all modes indicating the presence of free NH_2_ or aldehyde moieties (brown boxes) as well as imine linkages have completely vanished. Furthermore, several characteristic PBI modes (Figure 6 A, arrows) are present. Finally, TGA measurements (see the Supporting Information) revealed the presence of physisorbed H_2_O and high thermal stability (*T*_95 %_=584 °C, *T*_90 %_=659 °C). PXRD (see the Supporting Information) shows that PBI is completely amorphous only showing a broad and weakly pronounced halo at ≈15–35° (2*θ*, Cu‐K_α_). In terms of morphology, PBI from HTP contains some angular and sheet‐like particles of broad size distribution (≈20–300 μm, Figure 6 B) always covered by significantly smaller spherical particles of quite narrow size distribution (≈0.5–2.0 μm, Figure 6 B,C).

In summary, we have demonstrated here that in addition to the previously reported PIs and PA networks, HTP as an environmentally benign and experimentally simple strategy can also be used to obtain PPs and PBIs. While the major applications of PBIs lie in fireproof clothing, bearings, and fuel cell membranes, PPs are interesting for optical and electronic applications owing to their conjugated nature. Especially since HTP does not require any catalysts, the obtained products are very pure. Therefore, we decided to investigate the electrochemical properties of PP6, which was considered most promising because of the naphthalene moiety in its backbone.

### Electrochemical Characterization

**Figure 7:**
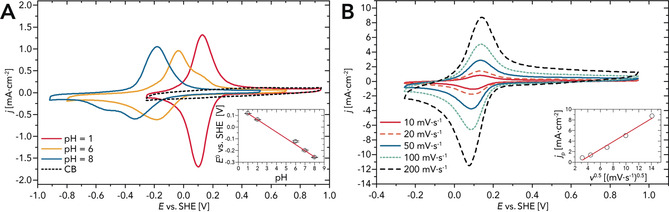
A) CV curves recorded with PP6‐carbon black electrodes in Ar‐saturated 0.1 m HClO_4_ (pH 1) and 0.2 m phosphate buffers (pH 6, and pH 8). Scan rate=20 mV s^−1^. The dashed line corresponds to the CV measured with CB (in 0.1 m HClO_4_). (inset) Redox potential (*E*_1/2_, measured by averaging the potentials of the anodic and cathodic peaks; see the Supporting Information) as a function of pH. The CV curves for pH 2 and pH 7 are reported in the Supporting Information. B) CV curves recorded with PP6‐carbon black electrodes in Ar‐saturated 0.1 m HClO_4_ at different scan rates. Inset: Randles–Sevcik plot of the anodic peak current density (*j*_p_) as a function of the square root of the scan rate.

PIs and quinone‐decorated polymers have recently been studied as aqueous battery electrodes in neutral and acidic electrolytes.^20^, ^21^, ^22^ While fabricating such a battery using PPs is way beyond the scope of this work, we decided to investigate PP6 from HTP by cyclic voltammetry (CV) in aqueous electrolytes as a first step. Figure 7 A shows the CV curves recorded with PP6‐carbon black (CB) electrodes at different pH values. In 0.1 m HClO_4_ (pH 1), the redox feature centered at ≈0.1 V vs. the standard hydrogen electrode (SHE) is reversible, as confirmed tby he symmetry of the cathodic and anodic waves and by the linear dependence of peak currents on the square root of the scan rate (inset of Figure 7 B). The peak separation (Δ*E*_p_) is 25 mV (see the Supporting Information for details). The most cathodic part of the CV is partly tilted because of the presence of the hydrogen evolution reaction, which is readily catalyzed both by CB and by PP6, especially under acidic conditions (*E* ${}_{2H{}^{+}/H{}_{2}}$ =−0.059 V vs. SHE at pH 1). We ascribe the redox feature to the reduction (and protonation)/ reoxidation (and deprotonation) of the PP6’s C=O moieties. Similar behavior was indeed observed for molecular quinones and quinone‐bearing polymers.^66^ At a scan rate of 10 mV s^−1^, the specific concentration of redox centers (as extrapolated from the charge transferred in the CV) is 3.2 mmol g^−1^. Based on the comparison with the nominal concentration of C=O groups in PP6 (4.9 mmol g^−1^), we infer that the reduction and protonation of PP6 (i.e., H^+^ intercalation) is not limited to the surface, but extends to the bulk, with a protonation efficiency of ≈70 % (see the Supporting Information). The position of the redox peak shifts using mild acidic and neutral electrolytes and the reversibility is partly lost (increase in Δ*E*_p_). The asymmetry and the overall changes in the electrochemical behavior may be caused by changes in the kinetics of the redox reaction at the C=O moiety, which have been demonstrated to vary depending on the nature of the reaction media in buffered and unbuffered electrolytes.^67^, ^68^ Moreover, it is plausible to expect that the redox behavior is given by the coexistence of the ion intercalation (K^+^ from buffer) and the protonation reaction. A 52 mV/pH unit slope (inset Figure 7 A) is observed when the shift of the redox potential is monitored (*E*_1/2_, calculated by averaging the potentials of the anodic and cathodic peaks, Table S1) as function of the pH of the electrolyte. This agrees with a predominant proton‐coupled redox reaction. While several redox polymers have been characterized in organic electrolytes,^20^, ^21^, ^69^, ^70^ to the best of our knowledge, there have been no studies on similar PPs in aqueous electrolytes. For PIs and quinone‐decorated polymers studied as aqueous battery electrodes, it was shown that they can be used for the intercalation of ions (Mg^+^, Na^+^). PP6 seems to possess similar properties, which we plan to explore in more detail in future studies.

## Conclusion

Herein, we demonstrate that in accordance with low‐molecular‐weight perinone it is possible to generate the pyrrone polymer PP6 (in the literature commonly known as BBB) hydrothermally starting from a suitable monomer salt precursor MS6 (synthesized from naphthalene bisanhydride and 3,3′‐diaminobenzidine). Upon elevating the reaction temperature, the degree of polymerization increases. MS6 and PP6 are morphologically virtually identical. Thus, a solid‐state transformation under shape retention occurring in dispersion is assumed. Yet, high‐temperature water must play a crucial role for facilitating this transformation, but its exact role still remains unclear. Furthermore, PP6 is electrochemically active in aqueous electrolytes. Under acidic conditions we ascribe the redox behavior, which was found to extend also to the bulk of PP6, to the reduction and protonation/reoxidation and deprotonation of the C=O moieties. This characteristic could be exploited, for example, in aqueous battery applications.

Interestingly, in the case of MS5 (a monomer salt synthesized from pyromellitic dianhydride and 3,3′‐diaminobenzidine), hydrothermal treatment does not directly yield the corresponding pyrrone polymer PP5. Instead, a polybenzimidazole intermediate with ‐COOH side groups (PBI‐COOH) is generated. Hence, the mechanistic pathway clearly differs from that of the hydrothermal polymerization towards PP6. The most efficient and effective way to entirely convert PBI‐COOH to fully condensed PP5 is a heat treatment at 400 °C. Regarding the morphology, no significant differences can be observed between PBI‐COOH and PP5, which is in agreement with a solid‐state transformation. However, they differ significantly from MS5. Therefrom, we propose a dissolution–polymerization–precipitation mechanism for the hydrothermal generation of PBI‐COOH. This lies in stark contrast to the hydrothermal polymerization towards PP6 where no intermediate dissolution phenomena are observed.

In contrast to PP6 and PP5, for pure, nonfunctionalized polybenzimidazole (PBI) a suitable monomer salt precursor could not be prepared. Nevertheless, the desired product can be generated hydrothermally by reacting terephthalaldehyde with 3,3′‐diaminobenzidine. Initially, an imine intermediate is formed under ambient conditions which can subsequently be converted to PBI hydrothermally.

We consider the successful preparation of these two classes of high‐performance polymers as a significant broadening of the scope of hydrothermal polymerization. These results are a promising starting point for future developments in the field. They open the door for a variety of new materials to be generated in a facile, experimentally simple, and environmentally benign way.

## Conflict of interest

The authors declare no conflict of interest.

## Supporting information

As a service to our authors and readers, this journal provides supporting information supplied by the authors. Such materials are peer reviewed and may be re‐organized for online delivery, but are not copy‐edited or typeset. Technical support issues arising from supporting information (other than missing files) should be addressed to the authors.

Supplementary
