# Supplementary material for: Hydrothermal Generation of Conjugated Polymers Using the Example of Pyrrone Polymers and Polybenzimidazoles
Source: Angew Chem Int Ed Engl. 2020 May 11;59(35):15050–60. doi: 10.1002/anie.202000367 (PMC7496105; doi:10.1002/anie.202000367)
Supplement: Supplementary file 1 — Supplementary [file ANIE-59-15050-s001.pdf]

Angewandte Chemie
Eine Zeitschrift der Gesellschaft Deutscher Chemiker

Supporting Information

Hydrothermal Generation of Conjugated Polymers Using the Example of Pyrrone Polymers and Polybenzimidazoles

*M. Josef Taublaender, Stefano Mezzavilla, Sophia Thiele, Florian Glöcklhofer, and Miriam M. Unterlass**

anie_202000367_sm_miscellaneous_information.pdf

Table of Contents

1 Methods 2
2 Chemicals 2
3 Experimental Details 3
3.1 PP6 3
3.1.1 Preparation of MS6 3
3.1.2 HTP of MS6 using a MW-autoclave 3
3.1.3 HTP of MS6 using a high-pressure autoclave 4
3.1.4 SSP of MS6 5
3.1.5 Fabrication of PP6 thin films 5
3.2 PP5 6
3.2.1 Preparation of MS5 6
3.2.2 HTP of MS5 using a MW-autoclave 6
3.2.3 Solid-state heat treatment of PBI-COOH to give PP5 7
3.3 PBI 8
3.3.1 HTP of TDA and DAB using a MW-autoclave 8
3.4 Electrochemical characterization 9
4 Analysis and Characterization 10
4.1 PP6 10
4.1.2 PXRD analysis after HT treatment for 7 days 10
4.1.2 ATR-FTIR analysis of samples obtained via SSP 10
4.1.3 DSC analysis 11
4.1.4 UV-Vis absorption spectrum of a PP6 solution 12
4.1.5 UV-Vis absorption spectrum of a PP6 thin film 12
4.1.4 Electrochemical characterization 13
4.2 MS5 14
4.2.1 ATR-FTIR and ^{1}H-NMR analysis 14
4.3 PP5 14
4.3.1 PXRD analysis of PBI-COOH and PP5 14
4.3.2 Mass loss during the transformation of PBI-COOH towards PP5 15
4.4 PBI 16
4.4.1 TGA measurement 16
4.4.2 PXRD analysis 16
5 References 17

1 Methods

Attenuated total reflectance Fourier transform infrared (ATR-FTIR) spectra were recorded on a Bruker Tensor 27 working in ATR MicroFocusing MVP-QL with a diamond crystal. Resolution was set to 4 cm^{-1} and spectra were recorded from 4000 cm^{-1} to 600 cm^{-1}.

^{1}H solution nuclear magnetic resonance (NMR) spectra were recorded on a Bruker AVANCE 250 spectrometer (250 MHz).

Powder X-ray diffraction (PXRD) data was collected with a PANalytical X'Pert Pro multipurpose diffractometer (MPD) in Bragg Brentano geometry operating with a Cu anode at 40 kV, 40 mA. An X-Celerator multichannel detector was used. Samples were ground and mounted as loose powders on silicon single crystal sample holders. The diffraction patterns were recorded between 5 ° and 60 ° (2θ) with 69.215 s/step and a step size of 0.0050134 °. Sample holders were rotated during the measurement with 4 s/turn.

Scanning electron microscopy (SEM) was carried out with a Quanta 200F FEI microscope. Typically, the samples were measured at 10 kV with a working distance of 7 - 9 mm and spot size 2.0. Prior to imaging, samples were loaded on carbon-coated stubs and coated by sputtering with a 17 nm thick layer of Au:Pd 60:40 alloy with a Quarum Q105T S sample preparation system.

Thermogravimetric analysis (TGA) was performed using a Perkin Elmer TGA 8000TM thermogravimetric analyser. All samples (5 – 7 mg) were transferred into alumina crucibles and heated from 30 °C to the desired target temperature under N_2-atmosphere at a heating rate of typically 2 or 10 °C·min^{-1}.

Differential scanning calorimetry (DSC) was carried out using a Netzsch STA 449 F3 Jupiter. After transferring approximately 5 mg of sample into an aluminum crucible, the sample was heated from 25 °C to 500 °C under N_2-atmosphere at a heating rate of 10 °C·min^{-1}.

Ultraviolet–visible (UV-Vis) absorption spectra were measured on a Jasco V-670 spectrophotometer in the wavelength region from 300 to 800 nm.

2 Chemicals

3,3'-Diaminobenzidine (DAB, >98%, TCI), naphthalene bisanhydride (NBA, >97%, TCI), pyromellitic dianhydride (PMDA, >97%, Sigma-Aldrich), terephthalic acid (TA, >97%, Sigma-Aldrich), terephthalaldehyde (TDA, >99 %, Sigma-Aldrich) and methanesulfonic acid (MSA, anhydrous, Sigma Aldrich) were purchased commercially and used as received.

3 Experimental Details

3.1 PP6

3.1.1 Preparation of MS6

In order to prepare MS6, 1.0000 g NBA (3.73 mmol, 1 eq.) was dissolved in 50 mL of 1 M NaOH under stirring at room temperature. Subsequently, conc. HCl was added dropwise while cooling in an ice bath until a pH of 1 was reached to ensure complete protonation of all carboxylic acid moieties of NTCA. Upon acidifying, a white solid precipitated which could be isolated *via* centrifugation. The yellowish liquid phase was removed and the remaining solid was centrifugated again in order to remove as much liquid and thus HCl as possible. The obtained, still wet NTCA was directly used for salt formation. Drying - even in a desiccator - would yield a crude product mixture containing NTCA-hydrates of unknown stoichiometry and partially formed mono- or bisanhydrides. Consequently, this would prevent an exact further working. Washing with H_2O or EtOH is disadvantageous as well due to partial dissolution of NTCA. After a third centrifugation step, NTCA was suspended in 750 mL of distilled H_2O while cooling in an ice bath. The obtained slurry was degassed by bubbling Ar through for 10 min. All further reaction steps were carried out under Ar atmosphere. Subsequently, finely ground 0.7191 g DAB (3.36 mmol, 0.9 eq.) was added to the cooled, degassed solution. After slowly warming up to room temperature, the obtained slurry was stirred overnight. The formed solid was isolated *via* vacuum filtration and washed thoroughly with distilled H_2O and EtOH. After drying in a desiccator, MS6 was obtained as a fine, purple powder.

Yield: quantitative

^{1}H-NMR (250.13 MHz, DMSO-d_6): δ [ppm]=8.00 (s, 4H, NTCA), 6.65 (s, 2H, DAB), 6.52 (m, 4H, DAB)

3.1.2 HTP of MS6 using a MW-autoclave

207 mg MS6 (c=0.01 mol·L^{-1}) were placed in a glass liner (V_{liner}=60 mL) and 40 mL of distilled H_2O were added. MS6 was initially dispersed at room temperature under intense stirring before the liner was placed in a PTFE-lined, stirred batch MW autoclave (V_{vessel}=120 mL). The dispersion was stirred magnetically while heating by MW irradiation to the intended reaction temperature T_R of 250 °C at a defined heating rate. The heating time t_H (time until T_R is reached; typically 10 min), reaction time t_R (time for which T_R is held constantly; typically 15 min) as well as certain deviations from the standard procedure are given for all experiments in Table S 1. At the end of the reaction, the autoclave was cooled back to room temperature *via* a stream of compressed air which took approximately 30 min. The obtained black, solid phase was isolated *via* vacuum filtration from the clear and translucent supernatant, washed with distilled H_2O and EtOH and dried in a vacuum oven at 80 °C overnight.

Yields: quantitative

The above-mentioned deviations from the standard procedure include:

- extension of t_R
- lowering of c
- extension of t_H
- variation of pH by adding HCl (aq.) or NH_3 (aq.)
- addition of HÜNIG's base (c=3 mmol·L^{-1}) to the dispersion of MS6

Table S 1: Conditions for HTP experiments to synthesize PP6 carried out in a stirred MW-assisted batch autoclave at 250 °C.

#	T_R [°C]	t_H [min]	t_R [min]	c [mol·L^{-1}]	other	ATR-FTIR comment
1	250	10	15	0.01	-	PP6, end groups visible
2	250	10	60	0.01	-	PP6, end groups visible
3	250	10	240	0.01	-	PP6, end groups visible
4	250	10	15	0.003	-	PP6, end groups visible
5	250	30	15	0.01	-	PP6, end groups visible
6	250	10	15	0.01	pH=4	PP6, end groups visible
7	250	10	15	0.01	pH=10	PP6, end groups visible
8	250	10	15	0.01	HÜNIG's base	PP6, end groups visible

3.1.3 HTP of MS6 using a high-pressure autoclave

129 mg MS6 (c=0.01 mol·L^{-1}) were placed in a quartz glass liner (V_{liner}=40 mL) and 25 mL of distilled H_2O were added. MS6 was initially dispersed at room temperature under intense stirring before the liner was placed in a non-stirred high-temperature high-pressure autoclave (HPA; V_{vessel}=80 mL). After applying an Ar-prepressure of 10 bar (in order to allow to reach T_Rs of more than 250 °C), the dispersion was heated *via* an external heating oven to the intended T_R (within this study the following T_Rs have been investigated: 250 °C, 275 °C, 300 °C, 325 °C and 350 °C). When using the HPA, t_H strongly depended on the target T_R. For example, heating to 250 °C took ≈ 35 min, whereas heating to 300 °C took ≈ 60 min and heating to 350 °C almost 120 min. After the desired t_R (typically 2 h and 12 h), the autoclave was quickly cooled back to room temperature by quenching in cold tap H_2O. The obtained black, solid phase was isolated *via* vacuum filtration from the clear and translucent supernatant, washed with distilled H_2O and EtOH and dried in a vacuum oven at 80 °C overnight.
The exact reaction conditions for all HTPs of MS6 towards PP6 using the HPA are listed in Table S 2.
Yields: quantitative

Note that the heating rates of the HPA (t_H≈35 min for reaching T_R=250 °C) were significantly lower than for the MW-assisted set-up (t_H≈10 min for reaching T_R=250 °C).

For checking whether re-subjecting PP6 to HT conditions for a longer time could help to improve crystallinity, 50 mg of PP6 prepared at T_R=350 °C and t_R=2 h were suspended in 40 mL of distilled H_2O in a quartz glass liner. Subsequently, the obtained black slurry was heated *via* the HPA to the desired T_R (250 °C and 300 °C, respectively) and kept under these conditions for 7 days.

Table S 2: Conditions for HTP experiments to synthesize PP6 carried out in a non-stirred high-pressure batch autoclaves at various T_Rs.

#	T_R [°C]	t_R [h]	c [mol·L^{-1}]	ATR-FTIR comment
1	250	0.25	0.01	incomplete, MS6 visible
2	250	2	0.01	PP6, end groups visible
3	250	12	0.01	PP6, end groups visible
4	275	2	0.01	PP6, end groups visible
5	275	12	0.01	PP6, end groups visible
6	300	2	0.01	PP6, end groups visible
7	300	12	0.01	PP6, end groups not visible
8	325	2	0.01	PP6, end groups visible
9	325	12	0.01	PP6, end groups not visible
10	350	2	0.01	PP6, end groups not visible

3.1.4 SSP of MS6

Approximately 15 - 20 mg of MS6 were heated under solvent free conditions from room temperature to the desired T_R (250 °C, 350 °C, 600 °C) at a heating rate of 10 °C·min^{-1} under N_2 atmosphere and held at T_R for a certain period of time. The exact reaction conditions for all SSPs of MS6 towards PP6 are listed in Table S 3.

Table S 3: Conditions for SSP experiments to synthesize PP6 carried out at various T_Rs.

#	T_R [°C]	t_R [h]	ATR-FTIR comment
1	250	2	mainly imide linkages and end groups visible
2	250	12	mainly imide linkages and end groups visible
3	350	2	partial double cyclization to PP6, imide and end groups visible
4	600	0.5	complete double cyclization to PP6, no imide, but end groups visible

3.1.5 Fabrication of PP6 thin films

Film fabrication was carried out according to a recent report.[1] Therefore, hydrothermally generated PP6 was dissolved in MSA (c=10 mg/mL) by stirring at room temperature for 24 h. Subsequently, the obtained dark red solution was spin coated on a glass substrate (static dispense, 2000 rpm, 30 s) and immersed in a methanol bath which immediately led to solidification of a thin film. The obtained film was dried in a desiccator at room temperature before annealing on a hot plate at 150 °C under a constant stream of N_2.

3.2 PP5

3.2.1 Preparation of MS5

In order to prepare MS5, 0.5726 g PMDA (2.63 mmol, 1.00 eq.) were suspended in 50 mL of distilled H_2O at room temperature and degassed by bubbling Ar through for 10 min. All further reaction steps were carried out under Ar atmosphere. Subsequently, the turbid, white suspension was heated to 80 °C and hold at this *T* until no solid was visible anymore indicating complete hydrolysis of PMDA to PMA. In the next step, 0.5456 g finely powdered DAB (2.55 mmol, 0.97 eq.) was added to the solution at 80 °C under stirring. The finally obtained slurry was stirred at 80 °C overnight. The formed solid was isolated *via* vacuum filtration and washed thoroughly with distilled H_2O and EtOH. After drying in a desiccator, MS5 was obtained as a fine, orange powder.

Yield: quantitative

^{1}H-NMR (250.13 MHz, DMSO-d_6): δ [ppm]=8.29 (s, 2H), 6.84 (s, 2H), 6.68 (s, 4H)

3.2.2 HTP of MS5 using a MW-autoclave

187 mg MS5 (c=0.01 mol·L^{-1}) were placed in a glass liner (V_{liner}=60 mL) and 40 mL of distilled H_2O were added. MS5 was initially dispersed at room temperature under intense stirring before the liner was placed in a PTFE-lined, stirred batch MW autoclave (V_{vessel}=120 mL). The dispersion was stirred magnetically while heating by MW irradiation to the intended T_R of 250 °C within a t_H of 10 min. The exact reaction conditions as well as certain deviations from the standard procedure are given for all experiments in Table S 4. At the end of the reaction, the autoclave was cooled back to room temperature *via* a stream of compressed air which took approximately 30 min. The obtained dark orange, solid phase was isolated *via* vacuum filtration from the clear and translucent supernatant, washed with distilled H_2O and EtOH and dried in a vacuum oven at 80 °C overnight. All products prepared from MS5 *via* HTP at 250 °C were identified as PBI-COOH intermediate. Full cyclization to PP5 had never been achieved.

The above-mentioned deviations from the standard procedure include:

- extension of t_R
- variation of pH by adding HCl (aq.) or NH_3 (aq.)
- addition of HÜNIG's base (c=3 mmol·L^{-1}) to the dispersion of MS5

Further attempts to achieve the direct HTP of fully condensed PP5 in the HPA (procedure in accordance to section 3.1.3) at a T_R of 300 °C led to decomposition of MS5.

Table S 4: Conditions for HTP experiments to synthesize PP5 carried out in a stirred MW-assisted batch autoclave at 250 °C.

#	T_R [°C]	t_H [min]	t_R [min]	c [mol·L^{-1}]	other	ATR-FTIR comment
1	250	10	15	0.01	-	no PP5, but PBI-COOH
2	250	10	60	0.01	-	no PP5, but PBI-COOH
3	250	10	240	0.01	-	no PP5, but PBI-COOH
4	250	10	15	0.01	pH=4	no PP5, but PBI-COOH
5	250	10	15	0.01	pH=10	no PP5, but PBI-COOH
6	250	10	15	0.01	HÜNIG's base	no PP5, but PBI-COOH

3.2.3 Solid-state heat treatment of PBI-COOH to give PP5

For obtaining fully condensed PP5, approximately 15 - 20 mg of PBI-COOH [generated *via* HTP of MS5 (c=0.01 mol·L^{-1}, T_R=250 ° C, t_H=10 min, t_R=15 min)] were heated under solvent free conditions from room temperature to 400 °C at a heating rate of 2 °C·min^{-1} under N_2 atmosphere and held at this T_R for 2 h. After cooling back to room temperature, PP5 was obtained as a black powder.

3.3 PBI

3.3.1 HTP of TDA and DAB using a MW-autoclave

53.7 mg TDA (0.4 mmol, 1 eq) and 85.7 mg DAB (0.4 mmol, 1 eq) were placed in a glass liner (V_{liner}=60 mL) and 40 mL of distilled H_2O (c=0.01 mol·L^{-1}) were added. The suspension of both comonomers was thoroughly stirred at room temperature which led to the precipitation of a red imine intermediate. Subsequently, the liner was placed in a PTFE-lined, stirred batch MW autoclave (V_{vessel}=120 mL). The dispersion was stirred magnetically while heating by MW irradiation to the intended T_R of 250 °C within a t_H of 10 min. At the end of the reaction, the autoclave was cooled back to room temperature *via* a stream of compressed air which took approximately 30 min. The obtained dark orange, solid phase was isolated *via* vacuum filtration from the clear and translucent supernatant, washed with distilled H_2O and EtOH and dried in a vacuum oven at 80 °C overnight.

The experiment using TA and DAB as comonomers was performed accordingly.

3.4 Electrochemical characterization

To prepare the electrode, a physical mixture (50/50 wt/wt) of polymer and carbon black (Cabot, Vulcan XC-72) was dispersed in a H_2O:isopropanol 80:20 (vol:vol) solvent mixture. The concentration of the suspension was set to 0.98 $mg{\cdot}mL^{-1}$. A diluted Nafion® perfluorinated ionomer (Sigma Aldrich, 5 wt% in lower aliphatic alcohols and water, contains 15-20% water) was added as binder to prevent detachment of the film from the substrate. The ionomer:(carbon+polymer) ratio was kept to 0.5 (wt/wt). The suspension was sonicated for ca. 5 min and drop-casted onto a 5 mm (0.196 cm^2) glassy carbon disk (HTW, Germany). The geometrical loading of the polymer was set to 50 $\mu g{\cdot}cm^{-2}$. The as-deposited ink was dried at room temperature under static air conditions.
The electrochemical characterization was carried out in a custom-made three-electrodes glass H-cell using an Ivium Vertex. One potentiostat (Alvatek) controlled by IviumSoft. The working electrode (WE) and CE compartments were separated by a Nafion® N117 proton conducting membrane, while the reference compartment was separated by a glass frit. A gold mesh (GoodFellow 99.9%) was used as counter electrode. The working electrode potential (E_{WE}) was referenced against a $Hg/HgSO_4$/sat. K_2SO_4 reference electrode (SI analytics). All the potentials are referred to the standard hydrogen electrode (SHE) scale according to:

$$E_{SHE}\,[V] = E_{WE} + E_{MSE}$$

where E_{WE} and E_{MSE} are the set potential at the WE (*vs.* $Hg/HgSO_4$) and the reference electrode potential (0.664 V ± 5mV *vs.* E_{SHE}), respectively. Electrochemical Impedance Spectroscopy (EIS) measurements (ΔV = 10 mV, from 30 kHz to 1 Hz) were carried out prior each measurement to determine the cell Ohmic resistance (*Ru*), which was corrected for *via* positive feedback (85% of *Ru*).
The cyclic voltammetry analysis was carried out in 0.1 M $HClO_4$ (Alfa Aesar, 70%, 99.9985% metals basis, pH = 1), 0.01 M $HClO_4$ (pH = 2) and a set of 0.2 M phosphate buffers (pH = 6, 7 and 8). The phosphate buffers were prepared from KH_2PO_4 (Sigma Aldrich, ACS reagent, ≥99.0%) and K_2HPO_4 (Sigma Aldrich, ACS reagent, ≥98.0%). The influence of the ionic strength on the activity coefficients of the ions was corrected for using the extended Debye–Hückel law. The pH of the buffers was verified to be 6 ± 0.1, 7 ± 0.1 and 8 ± 0.1 with a standard pH meter. All the electrolytes were saturated with Ar prior to each measurement.

4 Analysis and Characterization

4.1 PP6

4.1.2 PXRD analysis after HT treatment for 7 days

PXRD patterns of fully condensed and cyclized PP6 before (generated in HPA, c=0.01 mol·L^{-1}, T_R=350 °C, t_R=2h) and after HT treatment (re-subjection to HT conditions; T_R=300 °C, t_R=7 days) are shown in Figure S 1. Both patterns contain two broad features: one has its maximum at 11.6° (2θ, Cu-K$_\alpha$) (labeled as M1) and one exhibits its maximum at 25.8° (2θ, Cu-K$_\alpha$) (labeled as M2). Neither a significant sharpening nor the appearance of new reflections is observed. From these results, it becomes evident that subjecting PP6 to HT conditions for prolonged t_Rs does not influence product crystallinity.

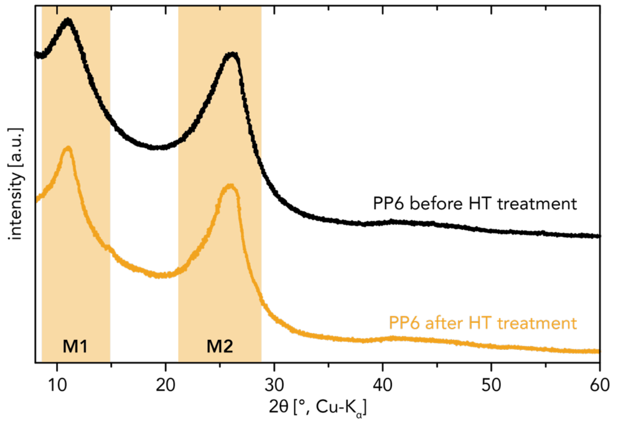

Figure S 1: PXRD patterns of PP6 before (generated in HPA, c=0.01 mol·L^{-1}, T_R=350 °C, t_R=2 h) and after heat treatment (HT conditions; T_R=300 °C, t_R=7 days): both patterns are virtually identical. Heat treatment does not influence crystallinity.

4.1.2 ATR-FTIR analysis of samples obtained *via* SSP

Figure S 2 A depicts the ATR-FTIR spectrum of partially condensed and cyclized PP6 synthesized through SSP at 350 °C for 2 h. Clearly, the spectrum shows the well-known anhydride end-group modes at ≈1740 cm^{-1} and ≈1780 cm^{-1} (highlighted by brown box). For further evaluation of the extent of reaction, in Figure S 2 B the ATR-FTIR spectra of different samples generated from MS6 *via* SSP at T_Rs of 250 °C (t_R=12h), 350 °C (t_R=2h) and 600 °C (t_R=0.5h) are shown. For the sample synthesized at 350 °C, the intensity of the anhydride end-group modes at ≈1740 cm^{-1} and ≈1780 cm^{-1} has clearly decreased compared to the sample generated at 250 °C. However, they are still rather intense (especially compared to hydrothermally obtained samples). Upon elevating T_R to 600 °C, a further decrease in the intensity of anhydride end-group modes can be observed. However, they never entirely vanish as it can be observed for samples generated *via* HTP (*e.g.* c=0.01 mol·L^{-1}, T_R=350 °C, t_R=2h).

Furthermore, it can be seen that the sample generated at 250 °C does not exhibit the characteristic C=O mode of PP6 at ≈1700 cm^{-1}. Instead, symmetric and asymmetric C=O stretching modes of a six-membered imide moiety at ≈1710 cm^{-1} and ≈1675 cm^{-1} can be observed. Increasing T_R to 350 °C leads to the appearance of the PP6 mode

at ≈1700 cm^{-1}. However, the imide C=O modes still remain present (as a shoulder of the very intense mode at ≈1700 cm^{-1}). Note that for reasons of better visibility in Figure S 2 B only the mode at ≈1675 cm^{-1} is highlighted by a brown box. A T_R of 600 °C enables full cyclization and therefore causes the imide modes to vanish entirely.

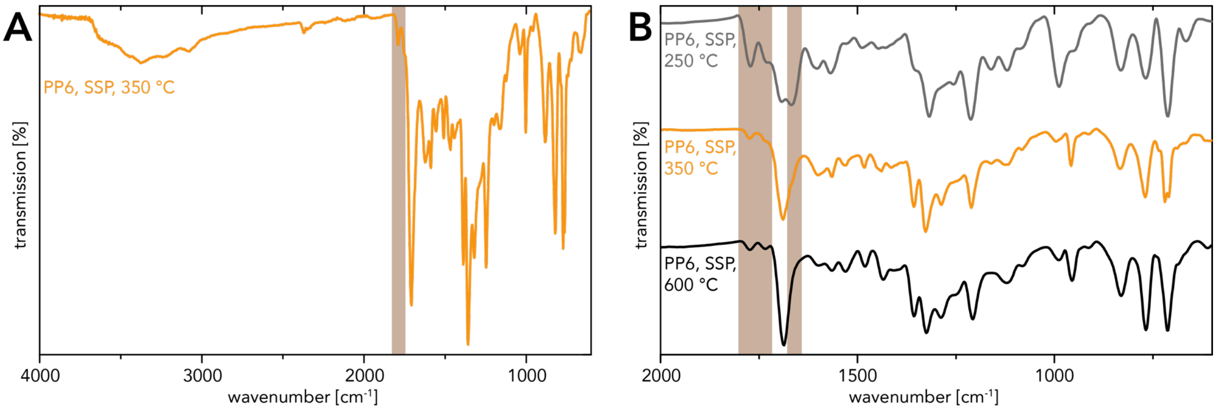

Figure S 2: **A** - ATR-FTIR spectrum of product obtained *via* SSP of MS6 at 350 °C (t_R=2 h). **B** – ATR-FTIR spectra of products obtained *via* SSP of MS6 at T_Rs of 250 °C (t_R=12h), 350 °C (t_R=2h) and 600 °C (t_R=0.5h). Modes that become less pronounced with increasing T_R, *i.e.* increasing extent of reaction, are highlighted by brown boxes.

4.1.3 DSC analysis

The DSC curve of a hydrothermally prepared PP6 sample showing no anhydride end-groups in the ATR-FTIR spectrum is depicted in Figure S 3. The sample had been dried at 100 °C prior to analysis in order to remove physisorbed H_2O. The absence of any peaks in the DSC curve indicates that no *T*-dependent structural changes occur upon heating PP6 prior to its decomposition.

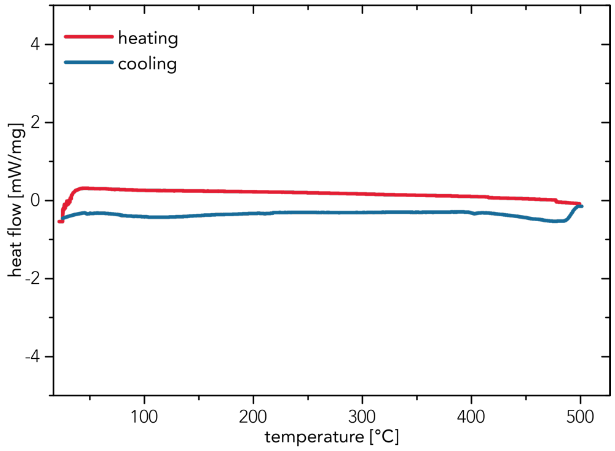

Figure S 3: DSC curve of PP6 (generated in HPA, c=0.01 mol·L^{-1}, T_R=350 °C, t_R=2 h) measured under N_2-athmosphere at a heating rate of 10 °C·min^{-1}.

4.1.4 UV-Vis absorption spectrum of a PP6 solution

For measuring solution UV-Vis absorption spectra, a solution of PP6 in MSA (c=0.002 mg/mL) was prepared. An exemplary spectrum of a PP6 sample that did not exhibit anhydride end-group modes in the ATR-FTIR spectrum is depicted in Figure S 4. It shows two intense absorption peaks with their maxima at 319 nm and 514 nm, respectively. These results agree well with the literature.[2]

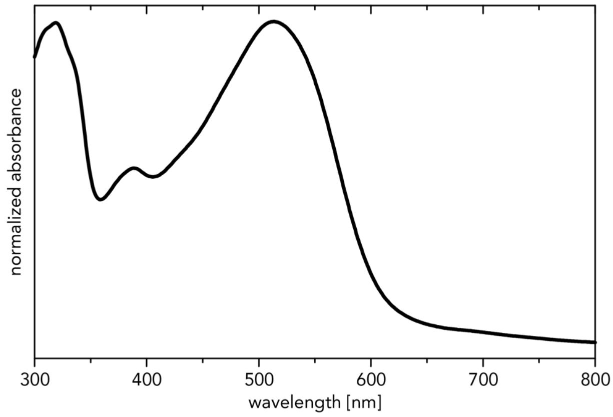

Figure S 4: UV-Vis absorption spectrum of hydrothermally synthesized PP6 (generated in HPA, c=0.01 mol·L^{-1}, T_R=350 °C, t_R=2 h) dissolved in MSA (c=0.002 mg/mL).

4.1.5 UV-Vis absorption spectrum of a PP6 thin film

PP6 thin films were prepared as described in subsection 3.1.5. The UV-Vis absorption spectrum of a film made from a PP6 sample that did not exhibit anhydride end-group modes in the ATR-FTIR spectrum is depicted in Figure S 5. It shows two intense absorption peaks with their maxima at 350 nm and 546 nm, respectively. These results agree well with the literature.[1,2]

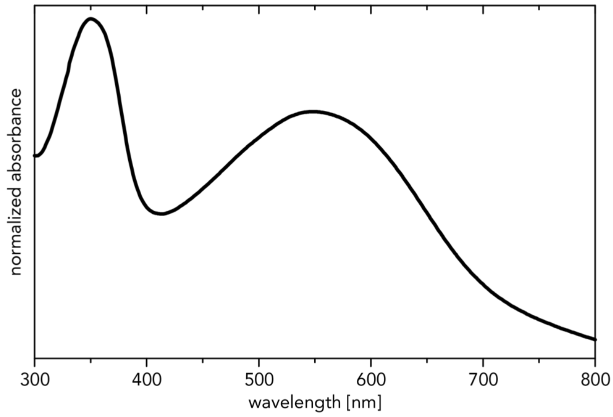

Figure S 5: UV-Vis absorption spectrum of PP6 (generated in HPA, c=0.01 mol·L^{-1}, T_R=350 °C, t_R=2 h) after fabricating a thin film.

4.1.4 Electrochemical characterization

Table S 5 lists redox potentials ($E_{1/2}$) and peak separations (ΔE_p) measured at different pH values. While ΔE_p increases with increasing pH, $E_{1/2}$ decreases.

Table S 6 depicts the estimation of the intercalation efficiency at different scan rates.

Figure S 6 shows the CV curves recorded with polymer-carbon electrodes in aqueous electrolytes (0.01 M $HClO_4$ (pH = 2) and 0.2 M phosphate buffers (pH = 7), respectively).

Table S 5: $E_{1/2}$ (measured by averaging the potentials of the anodic and cathodic peaks) and ΔE_p at different pH values.

pH (± 0.1)	$E_{1/2}$ vs. SHE [V]	ΔE_p [V]
1	0.115 ± 0.002	0.025
2	0.062 ± 0.003	0.067
6	-0.123 ± 0.006	0.150
7	-0.199 ± 0.005	0.130
8	-0.259 ± 0.003	0.155

Table S 6: Analysis of cyclic voltammetry measured with PP6 in Ar saturated 0.1M $HClO_4$ at different scan rates. Charge of redox peaks, corresponding concentration of redox species and intercalation efficiency. The carbonyl moieties were assumed as active redox species. Polymer electrode loading = 50 µg·cm^{-2}.

Scan rate [mV·s^{-1}]	Specific Charge [C·g^{-1}]		Redox centers concentration **(carbonyl)** [mmol·g^{-1}]			*Intercalation efficiency* [%]	
	cathodic	*anodic*	*cathodic*	*anodic*	*theoretical**	cathodic	anodic
10	306	346	3.2	3.6	4.9	65	74
20	250	290	2.6	3.0	4.9	53	62
50	220.8	228.8	2.3	2.4	4.9	47	49
100	194.4	215.4	2.0	2.2	4.9	41	46
200	166	181.8	1.7	1.9	4.9	35	39

* Calculated from the molecular weight of the repeating unit (410.392 g·mol^{-1}) hypothesizing a nominal concentration of two carbonyls per unit.

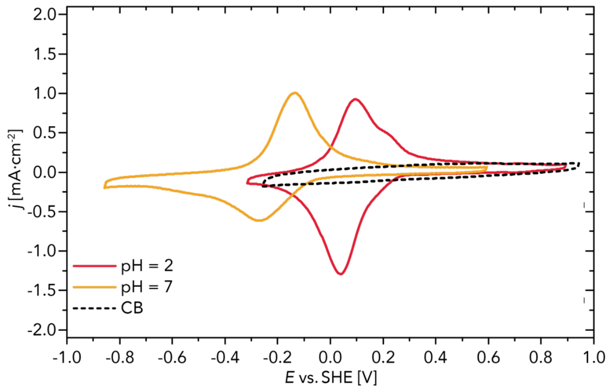

Figure S 6: CV curves recorded with polymer-carbon black electrodes in Ar saturated 0.01 M $HClO_4$ (pH = 2) and 0.2 M phosphate buffers (pH = 7). Scan rate=20 mV·s^{-1}. The dashed line corresponds to the CV measured with carbon black (in 0.1 M $HClO_4$).

4.2 MS5

4.2.1 ATR-FTIR and ^{1}H-NMR analysis

In the ATR-FTIR spectrum of MS5 depicted in Figure S 7 A several indicative modes confirming successful product formation ($\nu_{N-H}(NH_2)$≈3435 cm^{-1}; ≈3355 cm^{-1}; $\nu_{N-H}(NH_3^+)$≈2885 cm^{-1}; ≈2600 cm^{-1}; $\nu_{C=O}$(COOH)≈1690 cm^{-1}; $\nu_{C=O}(COO^-)$≈1500 cm^{-1}) are observed.

Figure S 7 B shows the ^{1}H-NMR spectrum of MS5 including the assignment of protons. The singlet (H_a) at 8.29 ppm corresponds to the aromatic protons of PMA, whereas the singlet (H_b) at 6.84 ppm and the broad singlet (H_c) at 6.68 ppm stem from the biphenylic protons. From the integral ratio H_a:H_b:H_c=2:2:4 one can conclude that MS5 is composed of DAB and NTCA in the desired 1:1 stoichiometry.

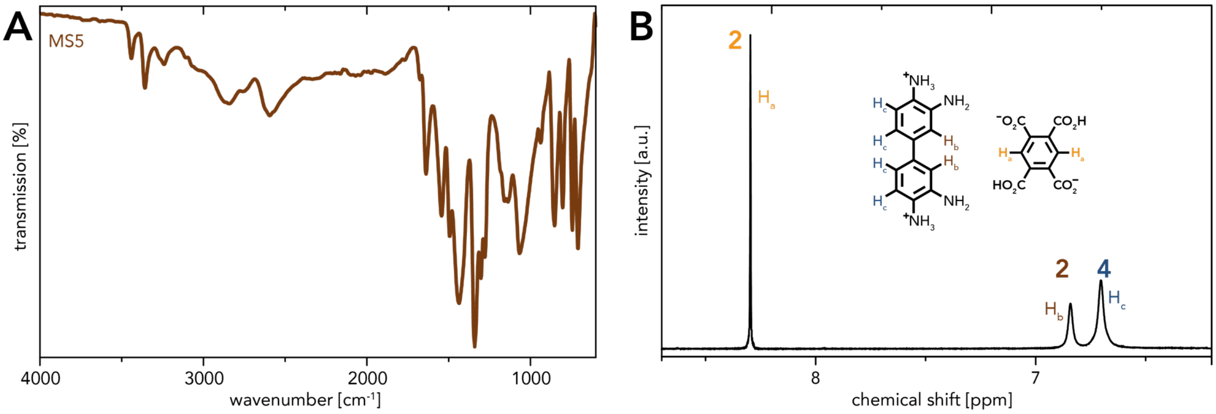

Figure S 7: Characterization of MS5: **A** – ATR-FTIR spectrum of MS5. **B** – ^{1}H-NMR spectrum of MS5 measured in DMSO-d_6: From the integral ratio H_a:H_b:H_c=2:2:4 one can conclude a 1:1 molar ratio of PMA:DAB in MS5.

4.3 PP5

4.3.1 PXRD analysis of PBI-COOH and PP5

PXRD patterns of PBI-COOH generated *via* MW-assisted HTP (c=0.01 mol·L^{-1}, T_R=250 °C, t_H=10 min, t_R=15 min) and PP5 synthesized *via* solid-state heat treatment of PBI-COOH (T_R=400 °C, t_R=2 h, N_2 atmosphere) are shown in Figure S 8. Both PXRD patterns only exhibit one broad and weakly pronounced halo having a maximum at approximately 26° (2θ, Cu-K_α), indicating that both species are amorphous and only show intermolecular π-stacking interactions between the polymer chains.

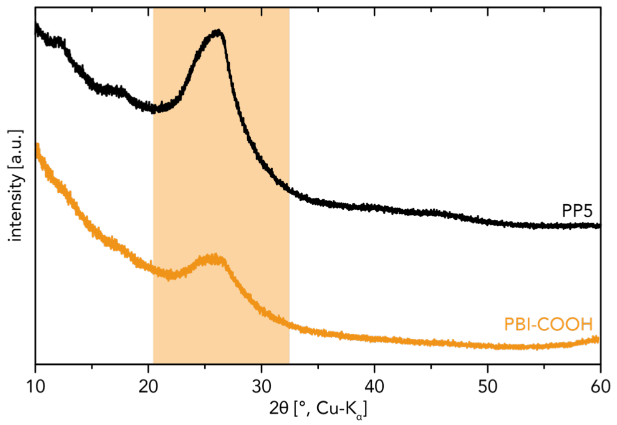

Figure S 8: PXRD patterns of PBI-COOH generated *via* MW-assisted HTP (c=0.01 mol·L^{-1}, T_R=250 °C, t_H=10 min, t_R=15 min) and PP5 synthesized *via* solid-state heat treatment of PBI-COOH (T_R=400 °C, t_R=2 h, N_2 atmosphere: both curves show broad features with a maximum around 26° (2θ, Cu-K$_\alpha$) (highlighted by orange box).

4.3.2 Mass loss during the transformation of PBI-COOH towards PP5

The theoretical mass loss Δm_{theo} caused by the thermal solid-state transformation of PBI-COOH towards PP5 due to the release of condensation H_2O can be calculated from the following equation:

$$\Delta m_{theo} = \frac{M_{PBI-COOH} - M_{PP5}}{M_{PBI-COOH}} \cdot 100 = \frac{2 \cdot M_{H_2O}}{M_{PBI-COOH}} \cdot 100$$

Δm_{theo} theoretical mass loss [%]

$M_{PBI-COOH}$ molecular weight of one repeating unit of PBI-COOH [g·mol^{-1}]; =396.36 g·mol^{-1}

M_{PP5} molecular weight of one repeating unit of PP5 [g·mol^{-1}]; =360.33 g·mol^{-1}

M_{H_2O} molecular weight of H_2O [g·mol^{-1}]; =18.02 g·mol^{-1}

According to this equation, a mass loss of 9.1 % is expected to occur for the transformation from PBI-COOH to PP5. *Via* TGA, the mass loss was experimentally determined to be 8.9 %. Together with ATR-FTIR analysis, this nicely confirms the successful transformation towards PP5.

4.4 PBI

4.4.1 TGA measurement

Figure S 9 depicts a representative TGA curve of hydrothermally synthesized PBI (MW-assisted synthesis, c=0.01 mol·L^{-1}, T_R=250 °C, t_H=10 min, t_R=15 min). The initial mass loss of 6 % stems from physisorbed H_2O. The characteristic polymer degradation temperatures are determined to be $T_{95\%}$=584 °C and $T_{90\%}$=659 °C.

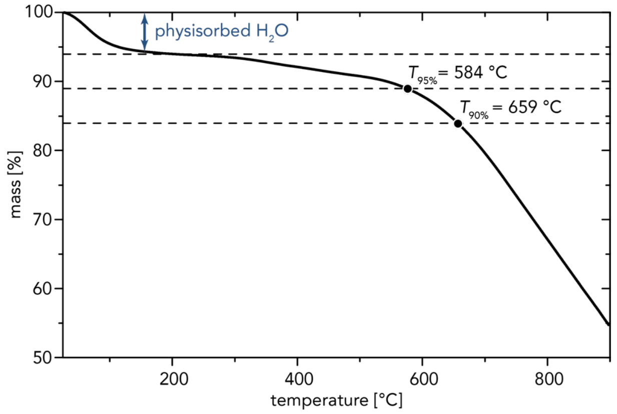

Figure S 9: TGA curve (in N_2, heating rate=10 °C·min^{-1}) of PBI generated from imine intermediate *via* stirred, MW-assisted synthesis (c=0.01 mol·L^{-1}, T_R=250 °C, t_H=10 min, t_R=15 min).

4.4.2 PXRD analysis

The PXRD pattern of PBI (MW-assisted synthesis, c=0.01 mol·L^{-1}, T_R=250 °C, t_H=10 min, t_R=15 min) shown in Figure S 10 evinces an amorphous sample. Only a broad halo ranging from 15° (2θ, Cu-K$_\alpha$) to 35° (2θ, Cu-K$_\alpha$) can be found.

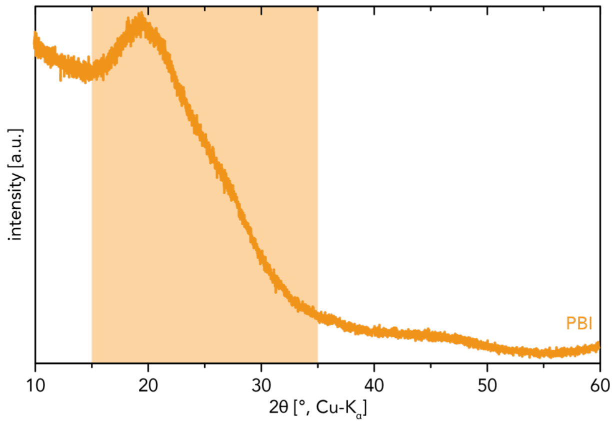

Figure S 10: PXRD pattern of PBI generated from imine intermediate *via* stirred, MW-assisted synthesis (c=0.01 mol·L^{-1}, T_R=250 °C, t_H=10 min, t_R=15 min): The only observed feature is an amorphous halo which is highlighted by an orange box.

5 References

[1] F. S. Kim, C. H. Park, Y. Na, S. A. Jenekhe, *Org. Electron.* **2019**, *69*, 301–307.
[2] M. F. Roberts, S. A. Jenekhe, *Polymer (Guildf).* **1994**, *35*, 4313–4325.
